# Riboflavin-photosensitized lipid oxidation in oil-in-water nanoemulsions: effects of oxygen scavengers and metal chelator

**DOI:** 10.3389/fnut.2026.1924218

**Published:** 2026-10-09

**Authors:** Fatma Bozanoglu, Sibel Uluata, Gokhan Durmaz

**Affiliations:** 1 Department of Food Engineering, Inonu University, Malatya, Türkiye; 2 Department of Nutrition and Dietetics, Inonu University, Malatya, Türkiye; 3 Department of Food Science, University of Massachusetts Amherst, Amherst, MA, United States

**Keywords:** emulsion, interface, photosensitized oxidation, prooxidant metals, riboflavin

## Abstract

This study examined how droplet size affects riboflavin-photosensitized lipid oxidation in oil-in-water emulsions and evaluated the roles of sodium azide (SA), superoxide dismutase (SOD), and ethylenediaminetetraacetic acid (EDTA) under light exposure. A model oil phase containing octadecane as the carrier oil and methyl linoleate as the oxidizable lipid substrate was used, while sodium dodecyl sulfate (SDS) served as a model anionic surfactant. Small- and large-droplet O/W (O/W) emulsions were produced by high-pressure homogenization at 20,000 psi and 1,500 psi, respectively and the emulsion was incubated 32 °C. The particle size of small and large droplets was determined 62.1 nm and 190 nm, respectively. The ζ-potential for small and large droplets was determined −73.3 and −90.8 mV. In the presence of riboflavin, light exposure markedly promoted lipid oxidation in both emulsion systems, as indicated by increased lipid hydroperoxide and headspace hexanal formation. The rate of lipid oxidation was higher in small-droplet emulsions than in large-droplet emulsions during photooxidation, suggesting that reduced droplet size increased oxidative susceptibility. However, SA did not significantly inhibit lipid hydroperoxide or hexanal formation, whereas SOD reduced lipid hydroperoxide accumulation but did not inhibit headspace hexanal formation. In contrast, EDTA strongly inhibited hexanal formation in both small- and large-droplet emulsions, indicating that transition metal-catalyzed hydroperoxide decomposition played an important role in secondary volatile oxidation. Overall, droplet size emerged as a key structural factor affecting riboflavin-photosensitized lipid oxidation in O/W emulsions, whereas metal chelation was particularly effective in limiting secondary volatile oxidation.

## 1 Introduction

Lipid oxidation is one of the main problems for the food industry because it causes negative changes in nutritional quality, flavor, and the appearance of food products and can also generate compounds associated with adverse effects on human health (1–3). In addition to bulk oils, many lipids are consumed in heterogeneous dispersed systems, such as dairy foods, beverages, desserts, yogurts, and sauces, and the droplet size of these products can range from 0.1 to 1,000 μm (4, 5). Lipid oxidation kinetics are different in bulk oil and heterogeneous lipid dispersion systems because different factors affect oxidation in these systems (4, 6, 7). Accordingly, understanding lipid oxidation in dispersed systems remains important for improving the oxidative stability of food emulsions (5, 8, 9). Recent reviews have also emphasized that lipid oxidation in emulsions is governed by complex interactions among emulsion structure, interfacial composition, prooxidants, antioxidants, and storage conditions (10, 11).

When lipids are dispersed as small particles in an aqueous-based food system, the oil–water interfacial area dramatically increases (5, 12). This enlarged interface can promote lipid oxidation because many oxidation reactions in O/W emulsions occur at or near the oil–water interface (13, 14). Under light exposure, the particle size of lipid droplets is also important because light transmission and optical properties of emulsions can influence their susceptibility to light-induced oxidation (13, 14). Uluata et al. (15) reported that the riboflavin-induced oxidation rate in fish O/W emulsions with smaller droplets was higher than that in emulsions with larger droplets. Similarly, Lethuaut et al. (16) found that coarse O/W emulsions had lower oxygen consumption than emulsions containing smaller droplets. More recently, ten Klooster et al. (17) demonstrated that lipid oxidation increased with decreasing droplet size in O/W emulsions, which they attributed to the increased contact area between lipids and continuous-phase prooxidants. However, some researchers reported that the formation of secondary oxidation products such as hexanal in emulsions with smaller droplets was lower than in larger-droplet emulsions (18). These contrasting findings indicate that the influence of droplet size on lipid oxidation is not universal and may depend on emulsion composition, oxidation conditions, and the oxidation marker evaluated.

Besides droplet size, other factors such as oxygen, photosensitizers, transition metals, light exposure, and interfacial properties also affect photooxidation in emulsion systems (9, 19–21). Due to their different electronic configurations, singlet oxygen is more reactive than triplet oxygen (22). Accordingly, controlling reactive oxygen species is an important strategy for limiting photosensitized oxidation in food systems (9, 22–25). Sodium azide is a strong physical quencher of singlet oxygen and is commonly used as a mechanistic probe to evaluate the involvement of singlet oxygen in photosensitized oxidation studies (20, 22). It has been reported that the presence of sodium azide in model oil systems decreases volatile compound formation due to singlet oxygen (26). In another study, milk containing riboflavin was stored under light at +4 °C, and the addition of sodium azide decreased the formation of hexanal, heptanal, and dimethyl disulfide, which were associated with singlet oxygen-mediated oxidation (27).

Superoxide anion is one of the reactive oxygen species and can be produced through Type I electron-transfer reactions during photosensitized oxidation (9, 24). In contrast, Type II photosensitized oxidation involves energy transfer to triplet oxygen, resulting in singlet oxygen formation (22, 23). Superoxide anion may also promote lipid oxidation by reducing transition metals and increasing their prooxidant activity. Superoxide dismutase (SOD) specifically dismutates superoxide anion and can be used as a mechanistic probe to evaluate the contribution of superoxide-associated reactions in food systems (20, 28).

Transition metal chelation is another important approach for inhibiting lipid oxidation because transition metals located in the aqueous phase can react with lipid hydroperoxides at or near the droplet surface, thereby promoting hydroperoxide decomposition and secondary oxidation. EDTA is a synthetic metal chelator and is widely used in the food industry (19, 29, 30). By chelating transition metals, EDTA can reduce metal-catalyzed hydroperoxide decomposition and the formation of secondary volatile oxidation products (20, 29, 30).

Lee and Decker investigated the effect of sodium azide, EDTA, and SOD in corn O/W emulsion systems during photosensitized oxidation and reported that EDTA inhibited lipid hydroperoxides and volatile compounds, whereas sodium azide only inhibited the formation of selected volatile oxidation products, such as 2-heptenal and 1-octen-3-ol (20). These findings suggest that the relative contribution of singlet oxygen, superoxide-associated reactions, and transition metal-catalyzed pathways may differ depending on the emulsion system and the oxidation marker evaluated.

Although previous studies have examined riboflavin-photosensitized oxidation, droplet-size effects, and the use of mechanistic probes in O/W emulsions, limited information is available on how singlet oxygen quenching, superoxide dismutation, and metal chelation influence lipid oxidation in emulsions with different droplet sizes. Thus, the aim of this study was to determine the effects of sodium azide as a singlet oxygen quencher, SOD as a superoxide-dismutating enzyme, and EDTA as a transition metal chelator on riboflavin-photosensitized lipid oxidation in small- and large-droplet O/W emulsions under light exposure.

## 2 Materials and methods

### 2.1 Materials

Octadecane (99% purity), methyl linoleate (99% purity), and riboflavin were purchased from Sigma-Aldrich (St. Louis, MO, USA). Sodium dodecyl sulfate (SDS), sodium azide (SA), ethylenediaminetetraacetic acid (EDTA), and superoxide dismutase (SOD) were also obtained from Sigma-Aldrich. All other reagents were of analytical or chromatographic grade.

### 2.2 Emulsion preparation

Octadecane (9.8 wt%) was melted by heating at 45 °C. The melted octadecane was mixed with methyl linoleate (0.2 wt%) and a 90% (w/w) aqueous solution containing 5 wt% SDS in 10 mM phosphate buffer (pH 7.0). To prepare a coarse emulsion, octadecane, methyl linoleate, and the aqueous solution were mixed at 45 °C using a hand-held high-speed blender (M133/1281–0, Biospec Products, Inc., Bartlesville, OK, USA) for 2 min. The coarse emulsion was then homogenized by passing it five times through a microfluidizer (M-110P, Microfluidics, Newton, MA, USA) at either 1,500 or 20,000 psi to obtain large- and small-droplet emulsions, respectively.

### 2.3 Particle size and ζ-potential measurement

The particle size distributions and droplet charge (ζ-potential) of the emulsions were measured using dynamic light scattering (Nano ZS, Malvern Instruments, Malvern, Worcestershire, UK). Samples were diluted 1:100 with 10 mM phosphate buffer solution (pH 7.0) to prevent multiple scattering. The particle size results were expressed as the Z-average particle diameter, while ζ-potential values were expressed in mV. Measurements were performed at 25 °C in triplicate.

### 2.4 Sample preparation for autooxidation and photosensitized oxidation tests

The auto-oxidation study, 1 mL of each nanoemulsion sample was placed in a 10 mL glass vial and sealed airtight. Sample vials were incubated at 32 °C in the dark. The photosensitized oxidation study, riboflavin (final concentration: 10 μM) was added to the nanoemulsion samples and mixed at 4 °C for 1 h in the dark. Samples without riboflavin were used as controls. EDTA (5 μM) was added to the nanoemulsion samples as a metal chelator. Sodium azide (SA; 10 μM) was added as a singlet oxygen quencher, and superoxide dismutase (SOD; 300 units/mg powder) was added to evaluate the contribution of superoxide-associated reactions in the emulsion system. Samples without EDTA, SA, and SOD were used as the corresponding control samples for the photosensitized oxidation study. Then, 1 mL of each sample was placed in a 10 mL glass vial and sealed airtight. Sample vials were stored under light exposure (1,440 lux) at 32 °C.

#### 2.4.1 Determination of lipid hydroperoxides formation

Lipid hydroperoxide formation in nanoemulsion samples was determined by the thiocyanate method as described by Uluata et al. (15), with slight modifications. Nanoemulsion samples (0.2 mL) were mixed with 1.5 mL of isooctane/2-propanol solution (3:1, v/v) and vortexed (three times for 10 s). The mixture was centrifuged at 3000 rpm for 10 min (CentrificTM Centrifuge, Thermo Fisher Scientific Inc., Fairlawn, NJ, USA). The upper organic layer (0.2 mL) was mixed with 2.8 mL of methanol/1-butanol solution (2:1, v/v), followed by the addition of 15 μL of 3.94 M ammonium thiocyanate and 15 μL of Fe^2+^ solution. The Fe^2+^ solution was freshly prepared from the supernatant of a mixture of equal volumes of 0.132 M BaCl₂ in 0.4 M HCl and 0.144 M FeSO₄. The solution was vortexed, held for 20 min at room temperature, and the absorbance was measured at 510 nm using a UV–Vis spectrophotometer (Genesys 20, Thermo Fisher Scientific Inc., Waltham, MA, USA). Hydroperoxide concentrations were determined using a standard curve prepared from cumene hydroperoxide.

#### 2.4.2 Determination of hexanal formation

Headspace hexanal was measured according to the method described by Uluata et al. (15) using a gas chromatograph (Model GC-2014, Shimadzu Co., Tokyo, Japan) equipped with a solid-phase microextraction (SPME) auto-injector (Model AOC-5000, Shimadzu Co., Tokyo, Japan). Nanoemulsions (1 mL) in 10 mL glass vials capped with aluminum caps and polytetrafluoroethylene (PTFE)/silicone septa were heated at 55 °C for 10 min in the autosampler heating block before measurement. A 50/30 μm divinylbenzene/carboxen/polydimethylsiloxane (DVB/CAR/PDMS) StableFlex SPME fiber (Supelco Co., Bellefonte, PA, USA) was then inserted into the vial headspace for 2 min to adsorb volatiles. The fiber was transferred to the GC injector port (250 °C) for 3 min. The injection port was operated in split mode, and the split ratio was set at 1:7. Volatiles were separated on a fused-silica capillary Equity-1 Supelco column (30 m × 0.32 mm i.d. × 1 μm) coated with 100% polydimethylsiloxane (PDMS) at 65 °C for 10 min. A flame ionization detector was used at 250 °C. Hexanal concentrations were determined from peak areas using a standard curve prepared from authentic hexanal. Each measurement was performed in triplicate, and results were expressed as mean values ± standard deviation.

### 2.5 Statistical analysis

Data were analyzed by one-way analysis of variance (ANOVA) using SPSS 20 (SPSS Inc., Chicago, IL, USA). Differences between mean values were compared using Duncan’s multiple range test at a significance level of *p* ≤ 0.05.

## 3 Results and discussion

### 3.1 Physical characterization of small- and large-droplet emulsions

The small-droplet emulsion was produced at 20,000 psi with five passes, and the particle diameter was measured as 62.1 ± 0.75 nm. The large-droplet emulsion was produced at 1500 psi with five passes, and the particle diameter was measured as 190.0 ± 1.02 nm (Figure 1). The clear difference in mean particle diameter confirmed the successful preparation of small- and large-droplet emulsions. The ζ-potential values of both emulsions were strongly anionic, with values of −73.3 ± 0.43 mV for the small-droplet emulsion and −90.8 ± 0.65 mV for the large-droplet emulsion, respectively. In addition to droplet size, the difference in ζ-potential indicates that the two emulsion systems differed in surface electrostatic properties. Therefore, the subsequent lipid oxidation behavior should not be interpreted solely as a droplet-size effect, but rather as the combined outcome of interfacial area and surface charge.

**Figure 1:**
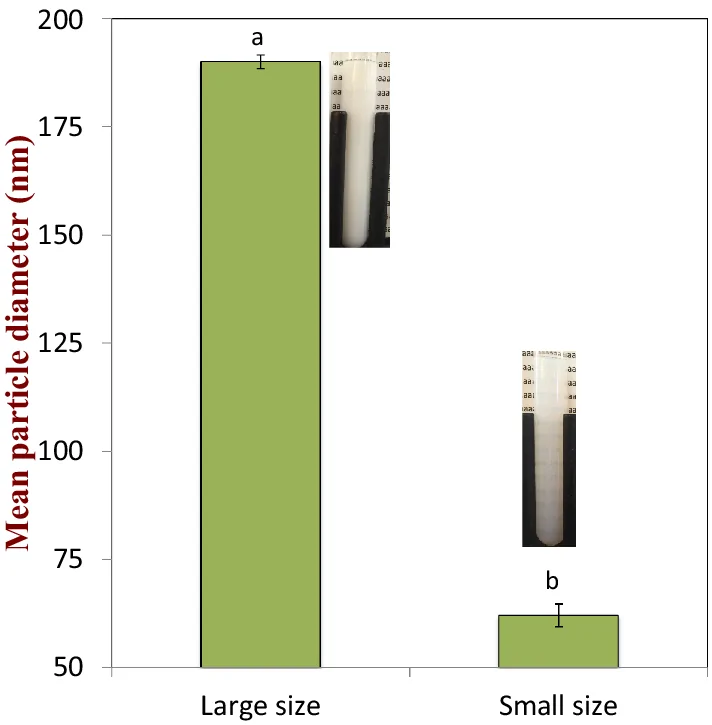
The mean particle diameter and visual appearance of small- and large-droplet emulsions. Values are expressed as mean ± standard deviation.

The appearance of the small-droplet emulsion was transparent, whereas the large-droplet emulsion was opaque (inset images in Figure 1). As expected, transparency increased as droplet size decreased, because smaller droplets scatter light less strongly than larger droplets.

Although both emulsions in the present study were within the nanoscale range, the smaller-droplet emulsion would still be expected to provide a larger oil–water interfacial area than the larger-droplet emulsion. Zhang et al. (31) reported that nanoemulsions possess an interfacial area approximately 4.85–4.94 times larger than that of coarse emulsions. This larger interfacial area implies greater contact between the oil phase and prooxidants present in the aqueous phase, thereby potentially promoting oxidation (31). Chen et al. (32) reported that physical properties such as droplet size, electrical charge (ζ-potential), and interfacial thickness significantly influence oxidation rates. Therefore, if higher oxidation is observed in the smaller-droplet emulsion, it should not be attributed only to increased interfacial area; differences in surface charge may also alter prooxidant–interface interactions (31, 32).

Based on these physical characteristics, it was hypothesized that differences in droplet size, light transmission, and surface electrostatic properties could influence the susceptibility of the emulsions to riboflavin-photosensitized oxidation. In addition, the responses to sodium azide, SOD, and EDTA could differ between small- and large-droplet emulsions because these probes interact with different oxidation pathways.

### 3.2 Riboflavin-photosensitized lipid oxidation

The formation of lipid hydroperoxides and headspace hexanal in small- and large-droplet emulsions under dark and light conditions is shown in Figures 2A,B, respectively. The emulsions were incubated at 32 °C under dark and light conditions to evaluate the formation of primary and secondary lipid oxidation products in the presence or absence of riboflavin. Samples without riboflavin were used as controls. In both smaller- and larger-droplet emulsions, lipid hydroperoxide levels of the control samples remained relatively unchanged during storage under both dark and light conditions, indicating limited auto-oxidation in the absence of riboflavin (Figure 2A).

**Figure 2:**
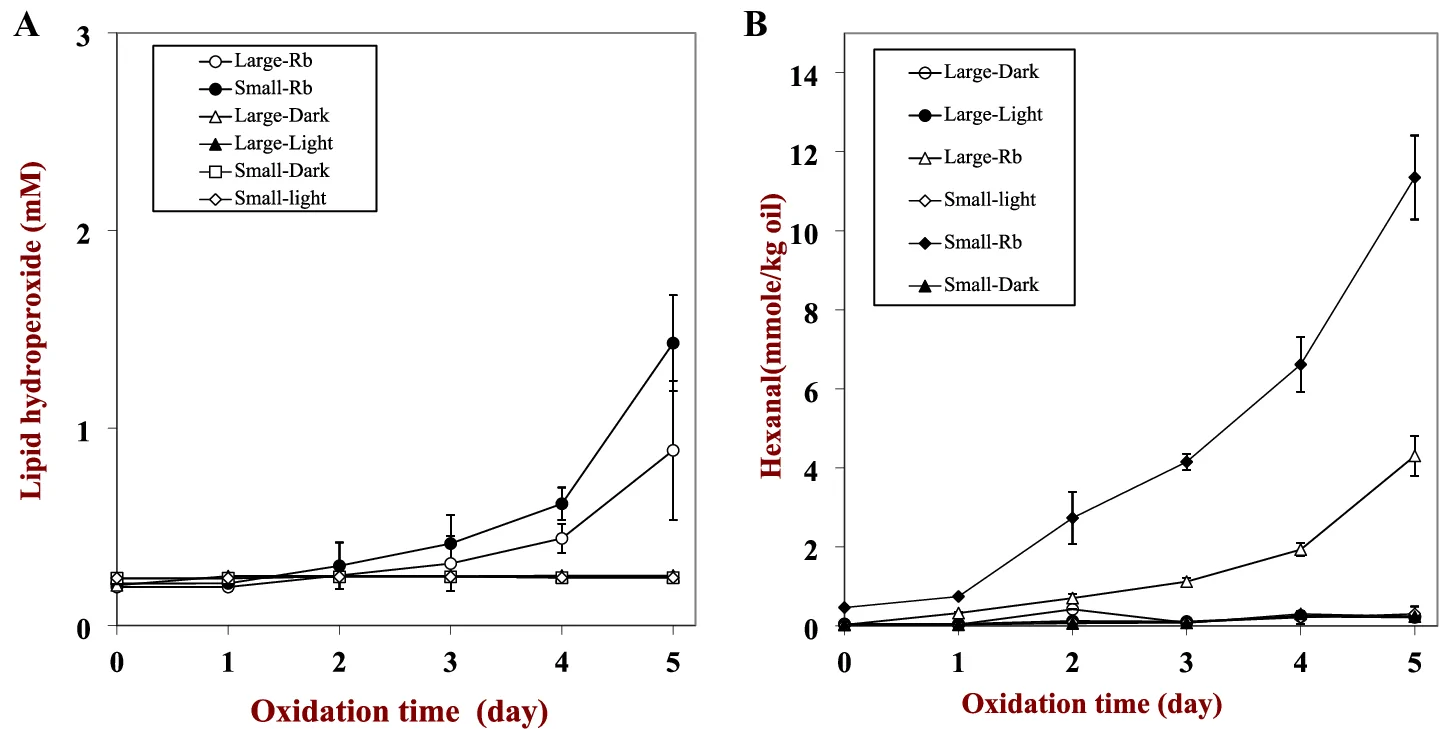
Lipid hydroperoxide **(A)** and Hexanal **(B)** formation in small- and large-droplet emulsions at 32 °C. Rb, riboflavin. Values are expressed as mean ± standard deviation.

In contrast, the presence of riboflavin markedly promoted lipid oxidation under light exposure. In small-droplet emulsions containing riboflavin, lipid hydroperoxide formation increased progressively during storage and became more pronounced after day 2 (Figure 2A). This pattern shows that riboflavin initiated primary lipid oxidation under illumination. A similar trend was observed for secondary oxidation, as headspace hexanal did not increase in the control samples under either dark or light conditions, whereas hexanal formation increased substantially in riboflavin-containing emulsions exposed to light (Figure 2B). Together, the increases in lipid hydroperoxides and hexanal show that riboflavin promoted both primary oxidation and the subsequent formation of secondary volatile oxidation products. Similar increases in lipid hydroperoxides and hexanal after light irradiation (with increases beginning 2 h after the onset of irradiation) have been reported in riboflavin-containing O/W emulsions, supporting the role of riboflavin in promoting photosensitized lipid oxidation (20).

The increase in oxidation products was more pronounced in small-droplet emulsions than in large-droplet emulsions. In particular, hexanal formation in small-droplet emulsions containing riboflavin increased rapidly after the first day of light exposure and continued to rise throughout storage, whereas in large-droplet emulsions, hexanal formation started to increase around day 2 and continued until day 5 (Figure 2B). These results indicate that riboflavin induced lipid oxidation in both emulsion systems, but its prooxidant effect was stronger in small-droplet emulsions.

The greater susceptibility of small-droplet emulsions to riboflavin-photosensitized oxidation may be attributed to their larger oil–water interfacial area, which increases the exposure of lipid substrates to riboflavin-derived reactive species generated in the aqueous phase under illumination. Since riboflavin is primarily located in the aqueous phase, the oil–water interface is expected to be a critical region where riboflavin-derived reactive species interact with lipid substrates. In small-droplet emulsions, the increased interfacial area may therefore facilitate the interaction between lipids and photosensitized reactive species. Consistent with this explanation, Uluata et al. (15) reported that lipid hydroperoxide formation at 37 °C was highest in emulsions containing only small droplets (*d* = 45 nm) and lowest in emulsions containing only large droplets (*d* = 216 nm). Moreover, at 20 °C, the formation rates of both lipid hydroperoxides and propanal increased as droplet size decreased, supporting the greater susceptibility of smaller droplets to riboflavin-induced oxidation (15). In addition, the difference in ζ-potential between small- and large-droplet emulsions suggests that surface electrostatic properties may also have contributed to differences in oxidation behavior by influencing the localization of prooxidants, metal ions, or reactive species at the interface. Therefore, the higher oxidation observed in small-droplet emulsions should be interpreted as the combined effect of increased interfacial area and altered surface electrostatic properties, rather than as a droplet-size effect alone. Previous studies have shown that droplet charge and oil–water interfacial properties can modulate lipid oxidation in emulsions by influencing the location and reactivity of prooxidants and antioxidants at the interface (11, 33).

### 3.3 Lipid hydroperoxide and hexanal formation

The simultaneous determination of lipid hydroperoxides and hexanal provided complementary information on the progression of lipid oxidation in the emulsions (Figures 2A,B). Lipid hydroperoxides are generally considered primary oxidation products that reflect the early stages of lipid oxidation, whereas hexanal is a secondary volatile oxidation product mainly formed through hydroperoxide decomposition (11, 34, 35). Therefore, these two markers should be interpreted as indicators of different but related stages of the oxidation process rather than as directly interchangeable measurements (11, 36).

As shown in Figures 2A,B, lipid hydroperoxide and hexanal formation followed different time-dependent patterns. In riboflavin-containing small-droplet emulsions, lipid hydroperoxide formation increased progressively during storage, whereas hexanal showed a more rapid and pronounced increase under light exposure (Figures 2A,B). This pattern indicates that primary oxidation products accumulated and were subsequently decomposed or further transformed into secondary volatile compounds. Thus, the rapid increase in both oxidation markers suggests that small-droplet emulsions were more susceptible not only to oxidation initiation but also to the progression of oxidation toward secondary volatile product formation. This interpretation is consistent with previous findings showing that smaller droplets can be more susceptible to riboflavin-induced oxidation in O/W emulsions (15).

In contrast, the delayed increase in hexanal formation in large-droplet emulsions may indicate a slower transition from primary oxidation to secondary volatile oxidation (Figure 2B). This delay may be associated with the lower interfacial area of large droplets, which could reduce contact between lipid substrates and riboflavin-derived reactive species. Previous studies have emphasized that the oil–water interface is a critical region for lipid oxidation in O/W emulsions and that droplet size can influence oxidation by modifying the interfacial contact between lipids and aqueous-phase prooxidants (8, 11, 15). Therefore, differences between lipid hydroperoxide and hexanal trends should not be interpreted as an inconsistency, but rather as evidence that hydroperoxide formation, hydroperoxide accumulation, and secondary volatile formation may proceed at different rates (11, 36). This distinction is important for interpreting the subsequent mechanistic probe experiments, particularly the EDTA treatment, where changes in lipid hydroperoxides and hexanal may reflect different effects on hydroperoxide formation and decomposition.

### 3.4 Effect of sodium azide on riboflavin-photosensitized oxidation

Sodium azide was used as a singlet oxygen quencher to evaluate the contribution of singlet oxygen-mediated oxidation to lipid hydroperoxide and hexanal formation, and the emulsions containing riboflavin but without sodium azide were used as control samples in this study (Figures 3A,B). Lipid hydroperoxide formation in all samples increased up to day 2 and then gradually decreased during the remaining storage period (Figure 3A). There were no significant differences between lipid hydroperoxide values in large- and small-droplet emulsions with or without sodium azide (*p* > 0.05). This pattern indicates that sodium azide did not statistically significant inhibit primary lipid oxidation in either emulsion system.

**Figure 3:**
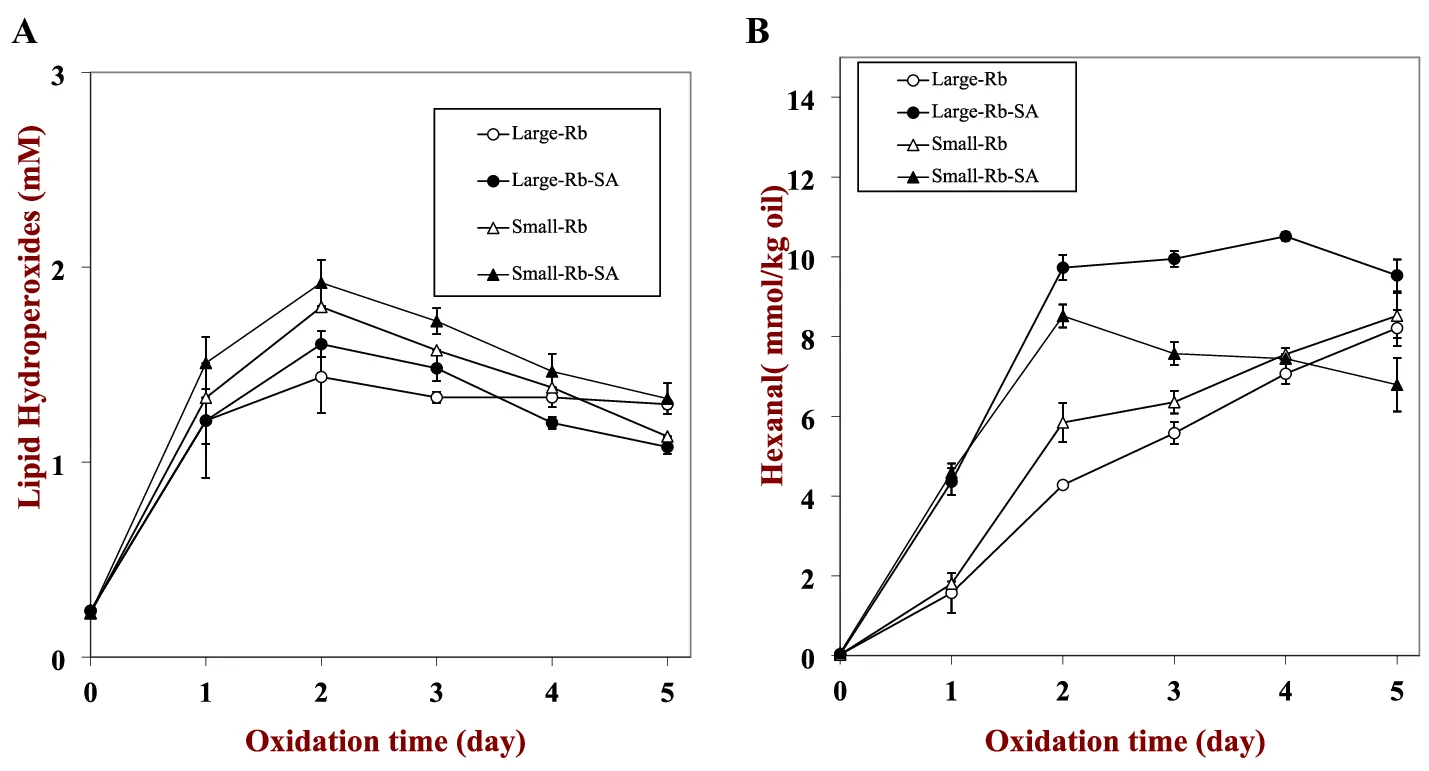
Lipid hydroperoxide **(A)** and Hexanal **(B)** formation in small- and large-droplet emulsions at 32 °C. Rb, riboflavin; SA, sodium azide. Values are expressed as mean ± standard deviation.

Hexanal formation in both control and sodium azide-containing emulsion samples started to increase on day 1 and continued throughout the storage period (Figure 3B). In some cases, hexanal values in sodium azide-containing emulsions were slightly higher than those of the corresponding control samples. However, these differences were not significant, indicating that sodium azide did not inhibit secondary volatile oxidation under the present conditions.

Sodium azide has been reported to reduce the formation of secondary oxidation products in bulk oil systems, where singlet oxygen-mediated oxidation may be more directly involved. In contrast, Lee and Decker reported that sodium azide did not decrease lipid oxidation products in riboflavin-photosensitized O/W emulsion systems (20). They suggested that other oxidation mechanisms may have proceeded faster than singlet oxygen generation. Similarly, in the present study, sodium azide did not significantly inhibit lipid hydroperoxide or hexanal formation in either large- or small-droplet emulsion systems therefore there was no statistically differences between samples (*p* > 0.05). Therefore, the lack of a significant sodium azide effect suggests that singlet oxygen-mediated oxidation made a limited contribution to lipid hydroperoxide and hexanal formation under the present experimental conditions (11, 20).

### 3.5 Effect of EDTA on riboflavin-photosensitized oxidation

The effects of the metal chelator EDTA on the oxidative stability of large- and small-droplet emulsions are shown in Figures 4A,B. Lipid hydroperoxide formation in both large- and small-droplet emulsions without EDTA increased markedly during storage, whereas EDTA reduced lipid hydroperoxide accumulation in both emulsion systems (Figure 4A). Although EDTA decreased lipid hydroperoxide levels, its inhibitory effect was particularly statistically evident for hexanal formation (*p* < 0.05).

**Figure 4:**
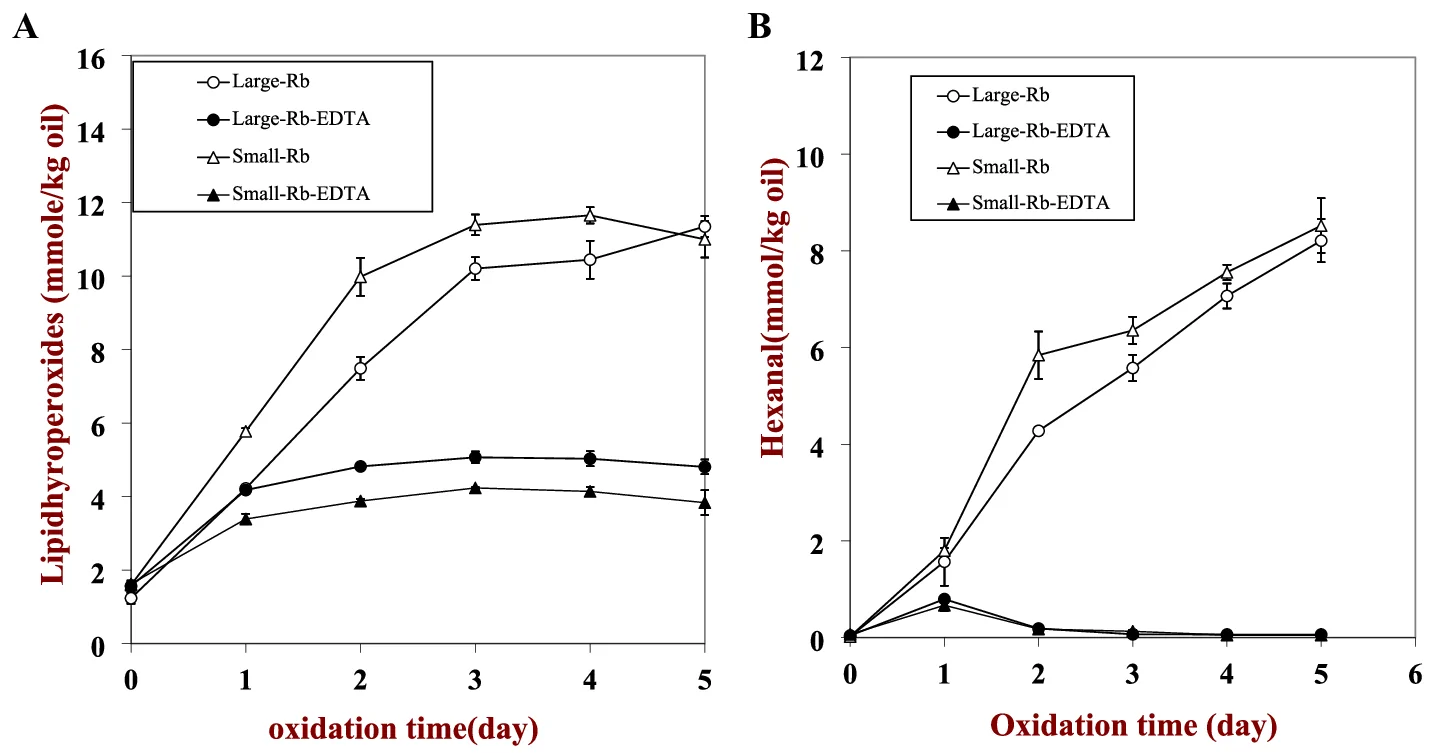
Lipid hydroperoxide **(A)** and Hexanal **(B)** formation in small- and large-droplet emulsions at 32 °C. Rb, riboflavin; EDTA, ethylenediaminetetraacetic acid. Values are expressed as mean ± standard deviation.

Hexanal formation in large- and small-droplet emulsions without EDTA increased sharply after day 1 and continued to rise throughout storage (Figure 4B). In contrast, EDTA strongly inhibited hexanal formation in both emulsion systems, with hexanal levels remaining very low during the oxidation period. This strong inhibition of hexanal formation suggests that EDTA mainly affected the decomposition of lipid hydroperoxides into secondary volatile oxidation products rather than only preventing the initial formation of hydroperoxides. Lipid hydroperoxides can be located near the oil–water interface of lipid droplets and may react with transition metals present in the surrounding aqueous phase. Therefore, chelation of transition metals is important for limiting lipid oxidation in O/W emulsions (8, 11). As a metal chelator, EDTA can reduce the catalytic activity of transition metals, thereby limiting metal-promoted hydroperoxide decomposition and the subsequent formation of volatile aldehydes such as hexanal (11, 20).

These data showed that EDTA inhibited lipid oxidation, with a particularly strong effect on secondary oxidation as reflected by hexanal formation. However, there were no statistically differences in the EDTA response between large- and small-droplet emulsion systems (*p* > 0.05). Therefore, the consistent inhibition of hexanal formation in both emulsion systems suggests that metal-catalyzed hydroperoxide decomposition was an important common pathway for secondary volatile oxidation, regardless of droplet size.

### 3.6 Effect of superoxide dismutase on riboflavin-photosensitized oxidation

Superoxide anion can be produced during riboflavin-photosensitized oxidation through Type I electron-transfer reactions, and SOD was used to dismutate superoxide anion radicals in this study (11, 20). Lipid hydroperoxide formation in large- and small-droplet emulsions increased during the early stages of incubation, reaching maximum levels around day 2, and then decreased during the remaining storage period (Figure 5A). Compared with the corresponding control samples, SOD-containing emulsions showed lower lipid hydroperoxide levels, particularly after day 2. However, this reduction was not accompanied by a decrease in hexanal formation (Figure 5B) (*p* < 0.05).

**Figure 5:**
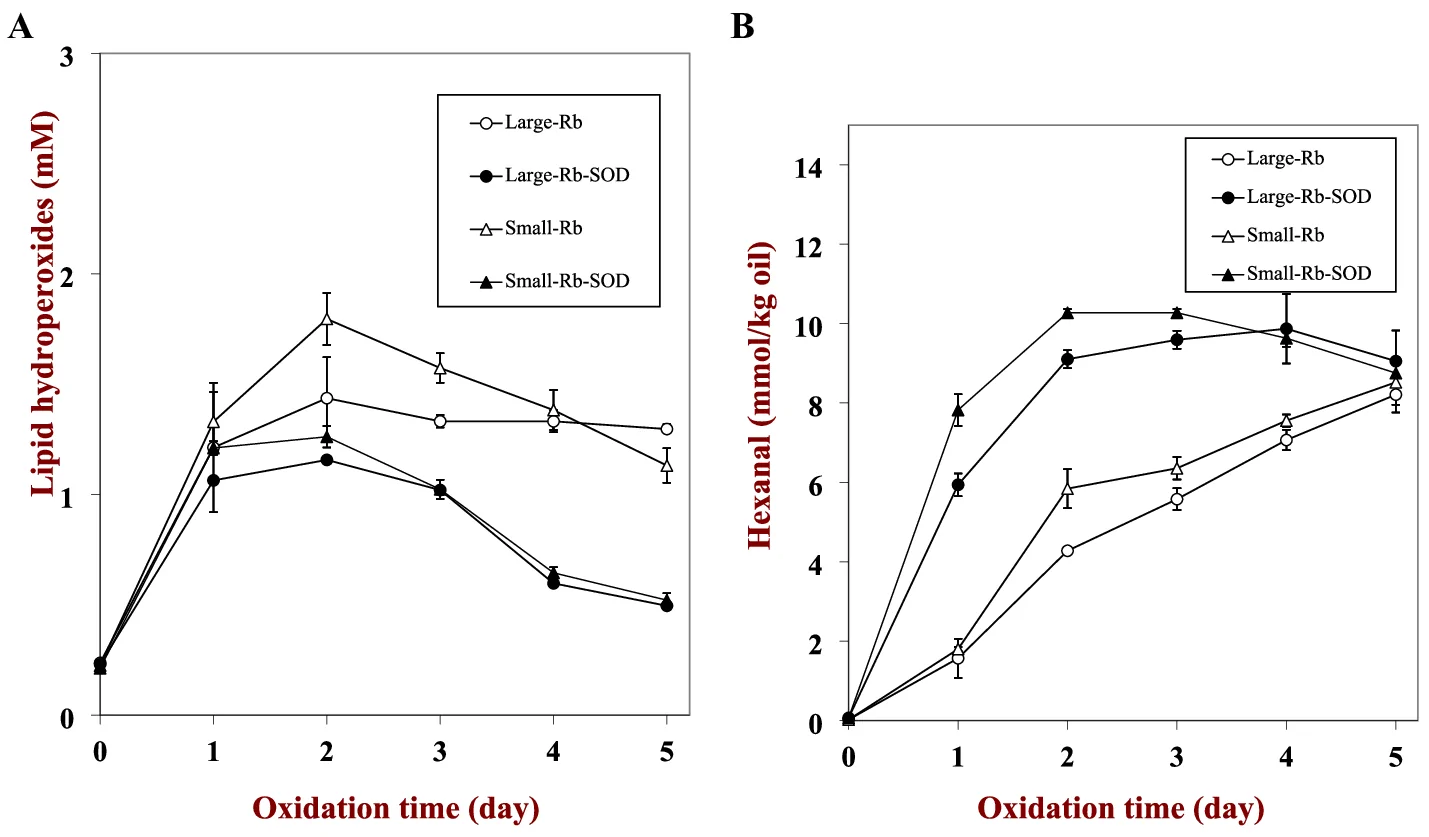
Lipid hydroperoxide **(A)** and Hexanal **(B)** formation in small- and large-droplet emulsions at 32 °C. Rb, riboflavin; SOD, superoxide dismutase. Values are expressed as mean ± standard deviation.

Hexanal formation increased in all riboflavin-containing emulsions during storage (Figure 5B). In contrast to the lipid hydroperoxide results, SOD did not inhibit hexanal formation in either emulsion system; rather, SOD-containing samples showed a more rapid increase in hexanal during the early stages of oxidation. Therefore, although SOD appeared to reduce lipid hydroperoxide accumulation, it did not inhibit the formation of secondary volatile oxidation products under the present conditions.

These results suggest that superoxide-associated reactions may have influenced lipid hydroperoxide accumulation, but they were not the dominant factor controlling hexanal formation. This response is consistent with the idea that lipid hydroperoxides and hexanal reflect different stages of lipid oxidation and may respond differently to mechanistic probes. Previous studies have also reported that SOD did not decrease lipid oxidation products in riboflavin-photosensitized O/W emulsions, possibly because other oxidative mechanisms proceeded more rapidly or dominated over superoxide-associated pathways (11, 20).

### 3.7 Comparation of SA, EDTA, and SOD effects

The different responses of the emulsions to SA, EDTA, and SOD provide useful mechanistic information on the dominant pathways involved in riboflavin-photosensitized lipid oxidation. SA did not significantly inhibit lipid hydroperoxide or hexanal formation, whereas SOD appeared to reduce lipid hydroperoxide accumulation but did not inhibit hexanal formation. In contrast, EDTA strongly inhibited hexanal formation in both small- and large-droplet emulsions. This contrast suggests that the measured oxidation products, particularly hexanal, were more strongly associated with EDTA-sensitive metal-catalyzed hydroperoxide decomposition than with sodium azide-sensitive singlet oxygen oxidation or SOD-sensitive superoxide-associated reactions (8, 11, 20).

The limited effect of SA indicates that singlet oxygen-mediated oxidation was not a major contributor to the measured oxidation products. Although SOD may have influenced lipid hydroperoxide accumulation, its lack of inhibition on hexanal formation suggests that superoxide-associated reactions were not the dominant pathway controlling secondary volatile oxidation. However, these results do not necessarily exclude the possible generation of singlet oxygen or superoxide-derived species during riboflavin photosensitization. Rather, they suggest that the contribution of these pathways to the measured oxidation products was limited, or that other oxidative reactions proceeded more rapidly in the emulsion systems. Similar observations were reported by Lee and Decker, who found that sodium azide and SOD did not effectively decrease lipid oxidation products in riboflavin-photosensitized O/W emulsions (20).

In contrast, the strong inhibitory effect of EDTA, especially on hexanal formation, indicates that transition metal-catalyzed reactions played an important role in secondary volatile oxidation. Transition metals can promote the decomposition of lipid hydroperoxides into alkoxyl and peroxyl radicals, leading to the formation of volatile aldehydes such as hexanal. Therefore, the stronger effect of EDTA on hexanal than on lipid hydroperoxides suggests that EDTA mainly limited hydroperoxide decomposition rather than completely preventing hydroperoxide formation (11, 20, 29).

Overall, the comparative responses of SA, EDTA, and SOD suggest that riboflavin-photosensitized oxidation in the present emulsion systems was controlled by multiple pathways, but these pathways did not contribute equally to primary and secondary oxidation products. While SA-sensitive singlet oxygen oxidation appeared to have limited influence on the measured markers, and SOD-sensitive reactions did not control hexanal formation, EDTA-sensitive metal-catalyzed hydroperoxide decomposition was more closely related to secondary volatile oxidation. This interpretation also supports the importance of evaluating both lipid hydroperoxides and hexanal, since primary oxidation product formation and secondary volatile product formation may respond differently to mechanistic probes.

### 3.8 Role of droplet size and ζ-potential in oxidation susceptibility

The higher lipid oxidation observed in small-droplet emulsions indicates that droplet size was an important factor controlling oxidative susceptibility in the present emulsion systems. Smaller droplets provide a larger oil–water interfacial area, which can increase the exposure of lipid substrates to aqueous-phase prooxidants, transition metals, and riboflavin-derived reactive species. Therefore, the greater formation of lipid hydroperoxides and hexanal in small-droplet emulsions may be attributed, at least in part, to the increased interfacial contact between lipid droplets and reactive species generated under light exposure. This interpretation is consistent with previous studies showing that smaller droplets can be more susceptible to lipid oxidation in O/W emulsions due to increased interfacial area and enhanced contact with continuous-phase prooxidants (8, 11, 15, 17).

In addition to droplet size, the difference in ζ-potential between small- and large-droplet emulsions indicates that the two emulsion systems also differed in surface electrostatic properties. Surface charge can influence the interfacial localization and reactivity of charged prooxidants, transition metal ions, and antioxidants in O/W emulsions. Thus, differences in ζ-potential may have contributed to the different oxidation behavior of small- and large-droplet emulsions by affecting the distribution of prooxidant species near the oil–water interface. Previous studies have shown that droplet charge and interfacial properties can modulate lipid oxidation in emulsions by altering electrostatic interactions between lipid droplets and prooxidants or antioxidants (8, 11, 19, 33).

Therefore, the higher oxidation observed in small-droplet emulsions should not be interpreted solely as a droplet-size effect. Rather, it may reflect the combined influence of increased oil–water interfacial area and altered surface electrostatic properties. The inhibitory effect of EDTA on hexanal formation further supports the importance of interfacial metal-related reactions, since transition metals located in the aqueous phase can interact with lipid hydroperoxides at or near the droplet interface and promote secondary volatile oxidation. Thus, droplet size and ζ-potential may influence oxidative stability not only by changing lipid exposure to riboflavin-derived reactive species, but also by affecting metal-catalyzed reactions at the oil–water interface (8, 11, 20, 29).

### 3.9 Implications for antioxidant strategies in emulsified food systems

From an applied food systems perspective, the greater susceptibility of small-droplet emulsions to riboflavin-photosensitized oxidation indicates that reducing droplet size may improve physical stability but can also increase oxidative vulnerability. Therefore, antioxidant strategies for emulsified foods should be designed together with emulsion structural properties, including droplet size, interfacial area, and surface electrostatic characteristics. Previous studies have emphasized that antioxidant efficacy in O/W emulsions depends not only on the chemical activity of antioxidants, but also on their location, interactions with the interface, and the distribution of prooxidants within the emulsion system (4, 10, 11).

The strong inhibition of hexanal formation by EDTA suggests that controlling transition metal-catalyzed hydroperoxide decomposition may be particularly important for reducing secondary volatile oxidation products. In the present study, SA did not significantly inhibit lipid hydroperoxide or hexanal formation, whereas SOD appeared to reduce lipid hydroperoxide accumulation but did not inhibit hexanal formation. In contrast, EDTA showed the most consistent inhibitory effect, especially on hexanal formation. This indicates that, under the present experimental conditions, metal chelation may be more effective than strategies targeting singlet oxygen- or superoxide-associated pathways alone, particularly for limiting secondary volatile oxidation. Thus, combining metal chelation with appropriate interfacial design may represent a useful strategy for improving the oxidative stability of riboflavin-containing O/W emulsions (4, 11, 20, 29).

The different responses of lipid hydroperoxides and hexanal also highlight the importance of monitoring both primary and secondary oxidation products. Measuring only lipid hydroperoxides or only volatile compounds may lead to an incomplete interpretation of oxidation development, particularly when antioxidants or mechanistic probes affect hydroperoxide formation and hydroperoxide decomposition differently. Therefore, the combined use of lipid hydroperoxides and hexanal provides a more comprehensive evaluation of oxidation progression and antioxidant performance in emulsified food systems (11, 35, 36).

## 4 Conclusion

In conclusion, riboflavin markedly promoted the formation of lipid hydroperoxides and hexanal in both emulsion systems under light exposure. Riboflavin-induced lipid oxidation was more pronounced in small-droplet emulsions than in large-droplet emulsions, most likely due to the larger oil–water interfacial area of smaller droplets and their greater exposure to riboflavin-derived reactive species under illumination. The difference in ζ-potential between the two emulsion systems further suggests that surface electrostatic properties may have contributed to the observed differences in oxidation behavior.

SA did not significantly inhibit lipid hydroperoxide or hexanal formation, suggesting that sodium azide-sensitive singlet oxygen oxidation was not the dominant pathway controlling the measured oxidation products under the present experimental conditions. SOD appeared to reduce lipid hydroperoxide accumulation but did not inhibit hexanal formation, indicating that superoxide-associated reactions were not the main pathway controlling secondary volatile oxidation. In contrast, EDTA reduced lipid hydroperoxide accumulation and strongly inhibited hexanal formation in both emulsion systems. The stronger effect of EDTA on hexanal formation indicates that transition metal-catalyzed hydroperoxide decomposition played an important role in the formation of secondary volatile oxidation products.

Overall, these findings show that oxidative stability in riboflavin-containing O/W emulsions is controlled by the combined effects of droplet size, interfacial properties, and metal-catalyzed reactions. Therefore, antioxidant strategies for such systems should not only target reactive oxygen species, but also control prooxidant metals through metal chelation and consider emulsion structural properties such as droplet size and oil–water interfacial characteristics.

## Data Availability

The raw data supporting the conclusions of this article will be made available by the authors, without undue reservation.

## References

[1] Frankel EN. Lipid oxidation: mechanisms, products and biological significance. J Am Oil Chem Soc. (1984) 61:1908–17. doi: 10.1007/BF02540830

[2] Frankel EN. Review†. Recent advances in lipid oxidation. J Sci Food Agric. (1991) 54:495–511. doi: 10.1002/JSFA.2740540402

[3] Liu JF Huang CJ. Tissue α-tocopherol retention in male rats is compromised by feeding diets containing oxidized frying oil. J Nutr. (1995) 125:3071–80. doi: 10.1093/JN/125.12.3071, 7500186

[4] Decker EA McClements DJ Bourlieu-Lacanal C Durand E Figueroa-Espinoza MC Lecomte J . Hurdles in predicting antioxidant efficacy in oil-in-water emulsions. Trends Food Sci Technol. (2017) 67:183–94. doi: 10.1016/J.TIFS.2017.07.001

[5] McClements DJ Decker EA. Lipid oxidation in oil-in-water emulsions: impact of molecular environment on chemical reactions in heterogeneous food systems. J Food Sci. (2000) 65:1270–82. doi: 10.1111/J.1365-2621.2000.TB10596.X

[6] Coupland JN McClements DJ. Lipid oxidation in food emulsions. Trends Food Sci Technol. (1996) 7:83–91. doi: 10.1016/0924-2244(96)81302-1

[7] Villeneuve P Bourlieu-Lacanal C Durand E Lecomte J McClements DJ Decker EA. Lipid oxidation in emulsions and bulk oils: a review of the importance of micelles. Crit Rev Food Sci Nutr. (2023) 63:4687–727. doi: 10.1080/10408398.2021.2006138, 34839769

[8] Berton-Carabin CC Ropers MH Genot C. Lipid oxidation in oil-in-water emulsions: involvement of the interfacial layer. Compr Rev Food Sci Food Saf. (2014) 13:945–77. doi: 10.1111/1541-4337.12097

[9] Choe E Min DB. Mechanisms of antioxidants in the oxidation of foods. Compr Rev Food Sci Food Saf. (2009) 8:345–58. doi: 10.1111/J.1541-4337.2009.00085.X

[10] Ghelichi S Hajfathalian M Yesiltas B Sørensen ADM García-Moreno PJ Jacobsen C. Oxidation and oxidative stability in emulsions. Compr Rev Food Sci Food Saf. (2023) 22:1864–901. doi: 10.1111/1541-4337.13134 36880585

[11] Hennebelle M Villeneuve P Durand E Lecomte J van Duynhoven J Meynier A . Lipid oxidation in emulsions: new insights from the past two decades. Prog Lipid Res. (2024) 94:101275. doi: 10.1016/J.PLIPRES.2024.101275, 38280491

[12] Chaiyasit W Elias RJ McClements DJ Decker EA. Role of physical structures in bulk oils on lipid oxidation. Crit Rev Food Sci Nutr. (2007) 47:299–317. doi: 10.1080/10408390600754248, 17453926

[13] McClements DJ. Advances in fabrication of emulsions with enhanced functionality using structural design principles. Curr Opin Colloid Interface Sci. (2012) 17:235–45. doi: 10.1016/J.COCIS.2012.06.002

[14] Waraho T Mcclements DJ Decker EA. Mechanisms of lipid oxidation in food dispersions. Trends Food Sci Technol. (2011) 22:3–13. doi: 10.1016/J.TIFS.2010.11.003

[15] Uluata S McClements DJ Decker EA. Riboflavin-induced oxidation in fish oil-in-water emulsions: impact of particle size and optical transparency. Food Chem. (2016) 213:457–61. doi: 10.1016/J.FOODCHEM.2016.06.103, 27451204

[16] Lethuaut L Métro F Genot C. Effect of droplet size on lipid oxidation rates of oil-in-water emulsions stabilized by protein. J Am Oil Chem Soc. (2002) 79:425–30. doi: 10.1007/S11746-002-0500-Z

[17] ten Klooster S Boerkamp VJP Hennebelle M van Duynhoven JPM Schroën K Berton-Carabin CC. Unravelling the effect of droplet size on lipid oxidation in O/W emulsions by using microfluidics. Sci Rep. (2024) 14:8895. doi: 10.1038/s41598-024-59170-9 PMC11024159 38632267

[18] Nakaya K Ushio H Matsukawa S Shimizu M Ohshima T. Effects of droplet size on the oxidative stability of oil-in-water emulsions. Lipids. (2005) 40:501–7. doi: 10.1007/S11745-005-1410-4, 16094860

[19] Donnelly JL Decker EA McClements DJ. Iron-catalyzed oxidation of menhaden oil as affected by emulsifiers. J Food Sci. (1998) 63:997–1000. doi: 10.1111/J.1365-2621.1998.TB15841.X

[20] Lee J Decker EA. Effects of metal Chelator, sodium Azide, and superoxide dismutase on the oxidative stability in riboflavin-photosensitized oil-in-water emulsion systems. J Agric Food Chem. (2011) 59:6271–6. doi: 10.1021/JF2001537, 21542578

[21] Lee JH Min DB. Analysis of volatile compounds from chlorophyll photosensitized linoleic acid by headspace solid-phase microextraction (HS-SPME). Food Sci Biotechnol. (2010) 19:611–6. doi: 10.1007/S10068-010-0086-Y

[22] Min DB Boff JM. Chemistry and reaction of singlet oxygen in foods. Compr Rev Food Sci Food Saf. (2002) 1:58–72. doi: 10.1111/J.1541-4337.2002.TB00007.X 33451241

[23] Huang R Choe E Min DB. Kinetics for singlet oxygen formation by riboflavin photosensitization and the reaction between riboflavin and singlet oxygen. J Food Sci. (2004) 69:C726–32. doi: 10.1111/J.1365-2621.2004.TB09924.X

[24] Montaña P Pappano N Debattista N Ávila V Posadaz A Bertolotti SG . The activity of 3- and 7-hydroxyflavones as scavengers of superoxide radical anion generated from photo-excited riboflavin. Can J Chem. (2011) 81:909–14. doi: 10.1139/V03-097

[25] Karogodina TY Sergeeva SV Stass DV. Stability and reactivity of free radicals: a physicochemical perspective with biological implications. Hemoglobin. (2011) 35:262–75. doi: 10.3109/03630269.2011.570187, 21599439

[26] Yang SO Lee JM Lee JC Lee JH. Effects of riboflavin-photosensitization on the formation of volatiles in linoleic acid model systems with sodium azide or D2O. Food Chem. (2007) 105:1375–81. doi: 10.1016/J.FOODCHEM.2007.05.002

[27] Lee JH Min DB. Changes of headspace volatiles in Milk with riboflavin photosensitization. J Food Sci. (2009) 74:C563–8. doi: 10.1111/J.1750-3841.2009.01295.X, 19895461

[28] Bancirova M. Sodium azide as a specific quencher of singlet oxygen during chemiluminescent detection by luminol and cypridina luciferin analogues. Luminescence. (2011) 26:685–8. doi: 10.1002/BIO.1296 21491580

[29] Cho YJ Alamed J McClements DJ Decker EA. Ability of chelators to alter the physical location and prooxidant activity of iron in oil-in-water emulsions. J Food Sci. (2003) 68:1952–7. doi: 10.1111/J.1365-2621.2003.TB07000.X

[30] Nuchi CD Julian McClements D Decker EA. Impact of tween 20 hydroperoxides and iron on the oxidation of methyl linoleate and salmon oil dispersions. J Agric Food Chem. (2001) 49:4912–6. doi: 10.1021/JF010370M, 11600043

[31] Zhang M Fan L Liu Y Li J. Effects of interface generation, droplet size and antioxidant partitioning on the oxidation rate and oxidative stability of water–in–oil emulsions: a comparison of coarse emulsions and nanoemulsions. Food Hydrocoll. (2023) 136:108227. doi: 10.1016/J.FOODHYD.2022.108227

[32] Chen B McClements DJ Decker EA. “Oxidation in different food matrices: how physical structure impacts lipid oxidation in oil-in-water emulsions and bulk oils”. In: Logan A, Nienaber U, Pan X, (eds), Lipid Oxidation: Challenges in Food Systems Champaing, IL, USA: (2013), p. 129–54. doi: 10.1016/B978-0-9830791-6-3.50006-0

[33] Mei L McClements DJ Decker EA. Lipid oxidation in emulsions as affected by charge status of antioxidants and emulsion droplets. J Agric Food Chem. (1999) 47:2267–73. doi: 10.1021/JF980955P, 10794621

[34] Ghorbani Gorji S Calingacion M Smyth HE Fitzgerald M. Comprehensive profiling of lipid oxidation volatile compounds during storage of mayonnaise. J Food Sci Technol. (2019) 56:4076–90. doi: 10.1007/S13197-019-03876-6, 31477979 PMC6706488

[35] Grebenteuch S Kroh LW Drusch S Rohn S. Formation of secondary and tertiary volatile compounds resulting from the lipid oxidation of rapeseed oil. Foods. (2021) 10:2417. doi: 10.3390/FOODS10102417/S1 PMC8535505 34681465

[36] Abeyrathne EDNS Nam K Ahn DU. Analytical methods for lipid oxidation and antioxidant capacity in food systems. Antioxidants. (2021) 10:1587. doi: 10.3390/ANTIOX10101587, 34679722 PMC8533275

